# Structural Insights into the Mechanism of Bacteriophage Mu Transposition

**DOI:** 10.1063/4.0001167

**Published:** 2025-10-27

**Authors:** Juhi Singh, Phoebe A Rice

**Affiliations:** 1 University of Chicago, Chicago, Illinois, USA; 2 University of Chicago, Chicago, Illinoise, USA

## Abstract

DNA transposons are mobile genetic elements that facilitate genomic rearrangements and have been widely studied for their roles in genome evolution and biotechnology [1,2]. Bacteriophage Mu employs the MuA transposase to catalyze DNA transposition through the formation of a higher-order nucleoprotein complex known as the transpososome [3,4].

We present a cryo-electron microscopy (cryo-EM) structure of the Mu transpososome in its post-integration state, resolved at 3.5 Å using full-length MuA and extended flanking host DNA. This structure represents a significant advancement over the previous crystal structure, which required truncation of the MuA C-terminal domain and removal of flanking host DNA to enable crystallization [4,5]. Notably, the cryo-EM map provides higher resolution in the catalytic domain, allowing for a more precise visualization of the active site architecture and the mechanism by which a single catalytic center mediates two successive transesterification reactions. A key insight from the current structure is the observed flexibility of domain IIIβ, which appears to play a critical role in accommodating and stabilizing DNA during strand transfer. This domain exhibits conformational variability that likely facilitates dynamic interactions with DNA. Additionally, the Mu end DNA is more flexible than the target DNA, which is held in a sharply bent U-turn conformation - a feature previously suggested by crystallographic and biochemical studies [4,5]. This contrast in flexibility provides insight into the conformational changes that occur during the transposition process. The flexibility at the Mu ends may facilitate transpososome assembly and allow precise positioning of the DNA ends into the active site for catalysis, while the relative rigidity of the target DNA may help to stabilize the final strand transfer complex.

Comparative analysis with the Tn7 transposase, particularly the recent cryo-EM structure of ShTnsB [6], reveals a conserved DDE catalytic core and similar domain organization, supporting a shared evolutionary origin. Both MuA and Tn7 transposases form transpososome complexes and catalyze DNA strand transfer via a transesterification mechanism. However, MuA functions as a single-component system with intrinsic catalytic and regulatory domains, while Tn7 requires multiple protein components for target site selection and integration [7,8]. The structural resemblance in the catalytic architecture explains their biochemical parallels, while differences in accessory domains and DNA engagement strategies account for their distinct regulatory mechanisms and target specificities[6,8].

